# Acute effects of maximal versus submaximal hurdle jump exercises on measures of balance, reactive strength, vertical jump performance and leg stiffness in youth volleyball players

**DOI:** 10.3389/fphys.2022.984947

**Published:** 2022-12-15

**Authors:** Raouf Hammami, Karim Ben Ayed, Manel Abidi, Hanen Werfelli, Amira Ajailia, Walid Selmi, Yassine Negra, Michael Duncan, Haithem Rebai, Urs Granacher

**Affiliations:** ^1^ Research Laboratory: Education Motor Skill Sports and Health (LR19JS01), Higher Institute of Sport and Physical Education of Sfax, University of Sfax, Sfax, Tunisia; ^2^ Higher Institute of Sport and Physical Education of Ksar Said, Manouba University, Tunis, Tunisia; ^3^ Research Unit (UR17JS01) “Sport Performance, Health and Society”, Higher Institute of Sport and Physical Education of Ksar Said, Manouba University, Tunis, Tunisia; ^4^ Centre for Sport, Exercise and Life Sciences, Coventry University, Coventry, United Kingdom; ^5^ Department of Sport and Sport Science, Exercise and Human Movement Science, University of Freiburg, Freiburg, Germany

**Keywords:** postural stability, stretch-shortening cycle exercise, youth, performance, volleyball

## Abstract

**Background:** Although previous research in pediatric populations has reported performance enhancements following long-term plyometric training, the acute effects of plyometric exercises on measures of balance, vertical jump, reactive strength, and leg stiffness remain unclear. Knowledge on the acute effects of plyometric exercises (i.e., maximal versus submaximal hurdle jumps) help to better plan and program warm-up sessions before training or competition.

**Objectives:** To determine the acute effects of maximal vs. submaximal hurdle jump exercise protocols executed during one training session on balance, vertical jump, reactive strength, and leg stiffness in young volleyball players.

**Materials and methods:** Thirty male youth volleyball players, aged 12–13 years, performed two plyometric exercise protocols in randomized order. In a within-subject design, the protocols were conducted under maximal (MHJ; 3 sets of 6 repetitions of 30-cm hurdle jumps) and submaximal (SHJ; 3 sets of 6 repetitions of 20-cm hurdle jumps) hurdle jump conditions. Pre- and post-exercise, balance was tested in bipedal stance on stable (firm) and unstable surfaces (foam), using two variables [center of pressure surface area (CoP SA) and velocity (CoP V)]. In addition, the reactive strength index (RSI) was assessed during countermovement maximal jumping and leg stiffness during side-to-side submaximal jumping. Testing comprised maximal countermovement jumps (CMJ).

**Results:** Significant time-by-condition interactions were found for CoP SA firm (*p* < .0001; d = 0.80), CoP SA foam (*p* < .0001; d = 0.82), CoP V firm (*p* < .0001; d = 0.85), and CoP V foam (*p* < .0001; d = 0.83). Post-hoc analyses showed significant improvements for all balance variables from pretest to posttest for MHJ but not SHJ. All power tests displayed significant time-by-group interactions for countermovement jumps (*p* < .05; d = 0.42), RSI (*p* < .0001; d = 1.58), and leg stiffness (*p* < .001; d = 0.78). Post-hoc analyses showed significant pre-post CMJ (*p* < .001, d = 1.95) and RSI (*p* < .001, d = 5.12) improvements for MHJ but not SHJ. SHJ showed larger pre-post improvements compared with MHJ for leg stiffness (*p* < .001; d = 3.09).

**Conclusion:** While the MHJ protocol is more effective to induce acute performance improvements in balance, reactive strength index, and vertical jump performance, SHJ has a greater effect on leg stiffness. Due to the importance of postural control and muscle strength/power for overall competitive performance in volleyball, these results suggest that young volleyball players should implement dynamic plyometric protocols involving maximal and submaximal hurdle jump exercises during warm-up to improve subsequent balance performance and muscle strength/power.

## Introduction

Balance stability, multidirectional locomotion, and jump related actions including spiking and blocking, are essential for success in volleyball (Sheppard et al., 2007; Ziv and Lidor, 2010). Given that spiking and blocking are associated with postural perturbations and require the maintenance of balance (Fuchs et al., 2020), production of power (Powers 1996 and Conditioning, 1996; Sheppard et al., 2007) under unstable conditions is needed to perform effectively. This is particularly the case in young players, where due to the need to jump maximally (e.g., when spiking) balance, muscle strength are important components of volleyball (Fuchs et al., 2020; Hammami et al., 2021). As a consequence, coaches have recommended the implementation of plyometric training in volleyball players’ training to improve balance and strength/power performance (Ziv and Lidor, 2010). This recommendation is evidence based given the scientific studies that demonstrate, in different populations, short-term effects of different jump training regimens on balance and jump performance (Lloyd et al., 2012; Dallas et al., 2020; Lauber et al., 2021; Werfelli et al., 2021). While research demonstrates that the acute effects of these exercises are similar in terms of performance, they differ with regards to the neural control mechanisms (Lees et al., 2004a; Lauber et al., 2021; Werfelli et al., 2021).

Plyometric training is a key aspect of conditioning in volleyball as it involves multi-joint actions and demands substantial muscular effort, primarily from the articulation of the ankle, knee, and the hip to perform vertical or horizontal jumps (Komi et al., 1987; Lees et al., 2004; Silva et al., 2019; Ramirez-Campillo et al., 2020). This can be achieved through maximal and/or submaximal plyometric exercises. Accordingly, this physical quality of unique mechanisms related to submaximal or maximal jumps needs to be differentially trained. For example, maximal jumps are characterized by the engagement of the hip extensor muscles (Lees et al., 2004a). During movement, the trunk muscles (i.e., transversus abdominus and multifidus) respond with anticipatory postural adjustments (APA) to the upper or lower limbs mobilization (Hodges and Richardson, 1996; Hodges and Richardson, 1998). Accordingly, it can be speculated that maximal plyometric exercises may enhance anticipatory postural adjustments and motor coordination by controlling landing forces and contributing to co-contraction of the involved musculature.

In addition, it has been shown that maximal plyometric exercises are needed to utilize contributions from the stretch-reflex (Voigt et al., 1998). In other words, the larger the stretch during landing, the greater the reflex contribution during take-off which ultimately results in maximal jump performance. Athletes who are more experienced with jump exercises may therefore show larger benefits from maximal versus submaximal plyometric exercises. Athletes with less PJT experience may favor submaximal exercises (Matic et al., 2015). Moreover, with regards to the training specificity concept (Behm and Sale, 1993), maximal jump exercises place a training stress on balance and stability. The increased negative speed during a maximal compared to a submaximal jump exercise increases the speed of pre-stretch of the knee extensors and the plantar flexors (Lees et al., 2004a). This subsequently decreases the delay between the pre-stretch and the concentric phase (Lees et al., 2004a) leading to an important mechanical output during the push-off phase and improvement of the sensitivity of afferent feedback pathways (Borghuis et al., 2008) leading to faster onset times of stabilizing muscles (Anderson and Behm, 2005). Therefore, jumping exercises, particularly conducted in maximal jumps, best define the requirement of the subsequent postural control and muscle-power activities (Lees et al., 2004a; Lloyd et al., 2012; Lauber et al., 2021). The longitudinal effects of jump training on balance and muscle power performance have been previously documented (Lloyd et al., 2012; Gebel et al., 2018; Hammami et al., 2021) but less is known about the acute effects of the different types of jump exercises on subsequent static balance and muscle power performance in youth.

Accordingly, the objective of this study was to investigate the acute effects of maximal (MHJ) versus submaximal (SHJ) hurdle jump exercise protocols, performed during a single training session on subsequent measures of balance [i.e., center of pressure surface area (CoP SA), velocity (CoP V)] and muscle strength/power [countermovement jump height (CMJ), leg stiffness, RSI] in young volleyball players. Based on the findings of similar studies (Lloyd et al., 2012; Dallas et al., 2020; Lauber et al., 2021; Moran et al., 2021), we hypothesized that the implementation of both MHJ and SHJ protocols would enhance balance and muscle strength/power in a sample of youth volleyball players. Given that the performance of plyometrics is task-specific (Voigt et al., 1998; Lees et al., 2004a), we speculated that maximal hurdle jump exercises would elicit the greatest changes in subsequent balance and muscle power measures in youth volleyball players.

## Materials and methods

### Participants

With reference to the study of Werfelli et al. (2021) on the acute effects of different plyometric and strength exercises on balance performance in youth weightlifters, an *a priori* power analysis, with a type I error rate of 0.05 and 80% statistical power, was computed. The analysis indicated that 24 participants would be sufficient to observe a significant interaction effect [effect size Cohen’s f = 0.3 for the center of presser velocity (CoP V) on unstable surface (i.e., BOSU ball with flat side facing up)]. Overall, 30 young and healthy volleyball players were enrolled in this study. All participants were members of a Tunisian team club (Esperance Sportive de Tunis) (Table 1) and practiced volleyball training for at least 3.4 years. In addition, players were familiar with plyometric exercises and volleyball specific exercises (i.e., blocks, smash) during competitions and training and had engaged in such for a minimum of 1 year before the start of the study. Data recorded for this study were taken during training. Legal guardians and participants provided written informed consent and assent after a thorough explanation of the objectives and scope of the research project, including the procedures, risks, and benefits of the study. The study was conducted according to the latest version of the Declaration of Helsinki, and the protocol was fully approved by the Local Ethics Committee before the commencement of the assessments. None of the participating athletes had a history of musculoskeletal, neurological, or orthopedic disorders that may have impaired their ability to execute the prescribed maximal and submaximal hopping exercise protocols and balance and power tests.

**TABLE 1:** Participant’s anthropometric and dynamic strength characteristics in the whole sample of male young volleyball players.

Variables	N = 30
Age (y)	12 ± 1
Height (cm)	161.5 ± 10.1
BM (kg)	50.5 ± 8.5
BF (%)	11.5 ± 2.2
MO (y)	−0.9 ± 0.9
APHV (y)	11.1 ± 1.9
TE	3.8 ± 1.3

Note: Table presented as means ± standard deviations. BM, body mass; BF, body fat; MO, maturity offset; APHV, age at peak height velocity; TE, training experience.

### Procedures

Two weeks prior to commencing the study, all subjects participated in a two familiarization sessions comprising the jumping exercise modalities and the strength, power, and balance tests to be used in subsequent sessions. The young volleyball players were instructed on how to perform the tests by a strength and conditioning specialist. Body height and mass were collected respectively. The sum of skinfolds (biceps, triceps, sub-scapular and supra-iliac) was also assessed. Body fat measurements were determined according to Deurenberg et al. (1990) and maturity offset was estimated using the Moore et al. (2015) equation. Following the warm-up, players completed all pretest balance and power protocols. Balance and power tests were randomly sequenced in the following order: balance, CMJ RSI and leg stiffness with approximately 2 min between 2 min rest between trials and tests. Thereafter, the athletes performed, in a randomized order, the two hurdle jump exercises protocols (SHJ and MHJ) on consecutive days, with at least 48 h between each. After the jump exercise intervention, all participants participated in post- tests. The applied jump protocols were performed in a random order after the warm-up as part of the participating players’ regular volleyball training regimens.

### Postural balance

Static balance performance was evaluated in bipedal stance with eyes opened on an unstable surface (i.e., BOSU ball with the flat side facing up) using a force plate with three strain gauges at a sampling rate of 40 Hz (PostureWin^©^, Techno Concept^®^, Cereste, France). The bipedal position was selected because the plyometric exercises were also performed in bipedal mode. To increase balance difficulty and to avoid a ceiling effect, the BOSU ball was put on top of the force plate. Participants were asked to stand as still as possible during testing with their arms comfortably placed downward at either side of the body; their bare feet were separated by an angle of 30° and their heels placed 5 cm apart. To maintain the same foot position for the balance assessment, a plastic device was used that allowed replication of the foot position. Throughout testing, participants were instructed to look straight ahead at a cross, placed at eye level on a nearby wall (2 m distance). Each test trial lasted 30 s. As dependent variables, two CoP sway parameters on firm and foam surface [(CoP SA firm, CoP SA foam) in mm^2^] and velocity [(CoP V Firm and CoP V Foam) in mm/ms] were analyzed. More specifically, CoP V indicates the total distances covered by the CoP divided by the duration of the sampled period and CoP SA represents the ellipse of the area covered by the trajectory of the CoP (Schubert and Kirchner, 2014). For these parameters, the lower the value, the better the balance performance (Caron et al., 2000). Intraclass correlation coefficients (ICCs) are presented in Table 2 for the respective CoP parameters.

**TABLE 2:** Test-retest reliability of the applied balance and strength/power tests.

Variables	ICC (95% CI)	SEM	CV (%)
Balance			
CoP SA firm	0.89 [0.81–0.90]	1.32	0.75
CoP SA foam	0.88 [0.78–0.95]	1.47	0.86
CoP V firm	0.89 (0.78–0.91)	0.97	2.17
CoP V foam	0.81 [0.72–0.86]	7.81	1.43
Muscle power			
CMJ	0.91 [0.86–0.93]	2.12	1.71
RSI	0.89 [0.85–0.90]	2.28	2.39
Leg stiffness	0.87 (0.81–0.90)	1.74	2.24

Notes: Values are means and standard deviations (SD), ICC, intra-class correlation coefficient; CV, coefficient of variation, CoP SA, firm center of pressure surface area on firm surface; CoP SA, foam center of pressure surface area on foam surface; CoP V, firm center of pressure velocity on firm surface; CoP V, foam center of pressure velocity on firm surface.

### Muscle power

As proxies of muscle power, the countermovement jump (CMJ) and maximal as well as submaximal hopping tests were applied. All of these tests proved to be reliable and valid in pediatric populations (Lloyd et al., 2011; Hammami et al., 2016). During testing, players landed in the same position and location as they started the test to minimize horizontal displacement and influence on flight time. All vertical jump tests were performed using an Ergojump^®^ system 148 (Ergojump apparatus, Globus Italia, Codogne, Italy), which recorded jump height, contact and flight times. Each test was separated by a passive recovery period of at least 5 min.

The CMJ involved the participants to lower the center of mass as quickly as possible from an upright standing position to a self-selected knee angle which was immediately followed by maximal acceleration of the center of mass in vertical direction. Two trials were performed with approximately a 2 min recovery and the best result was used for analysis. Maximal and submaximal hopping protocols were performed in the same manner as previously described (Lloyd et al., 2011; Hammami et al., 2016). The maximal hopping protocol involved participants performing five repeated bilateral maximal vertical hops in place on the contact mat. Participants were instructed to maximize jump height and minimize ground contact time (Lloyd et al., 2011). The first jump in each trial was not included in the analysis. Jump height and ground contact time were averaged across the four remaining hops and used to calculate the reactive strength index as follows: RSI = Jump height (m)/ground contact time (s).

Leg stiffness was measured during submaximal hopping by modeling the vertical ground reaction force, based on the flight and contact time during hopping (Dalleau and Allard, 2009; Lloyd et al., 2011). Participants were asked to hop bilaterally for 20 consecutive hops at 2.0 Hz. An electronic metronome helped the subjects to maintain the required frequencies by means of an auditory signal. The initial five and last five hops were discarded and the subsequent 10 hops averaged to provide a measure of leg stiffness. Leg stiffness (kilo Newtons per meter) was calculated from the average contact time and flight time across the 10 hops, together with body mass (Dalleau and Allard, 2009).

### Hurdle jump exercise protocols

**FIGURE 1:**
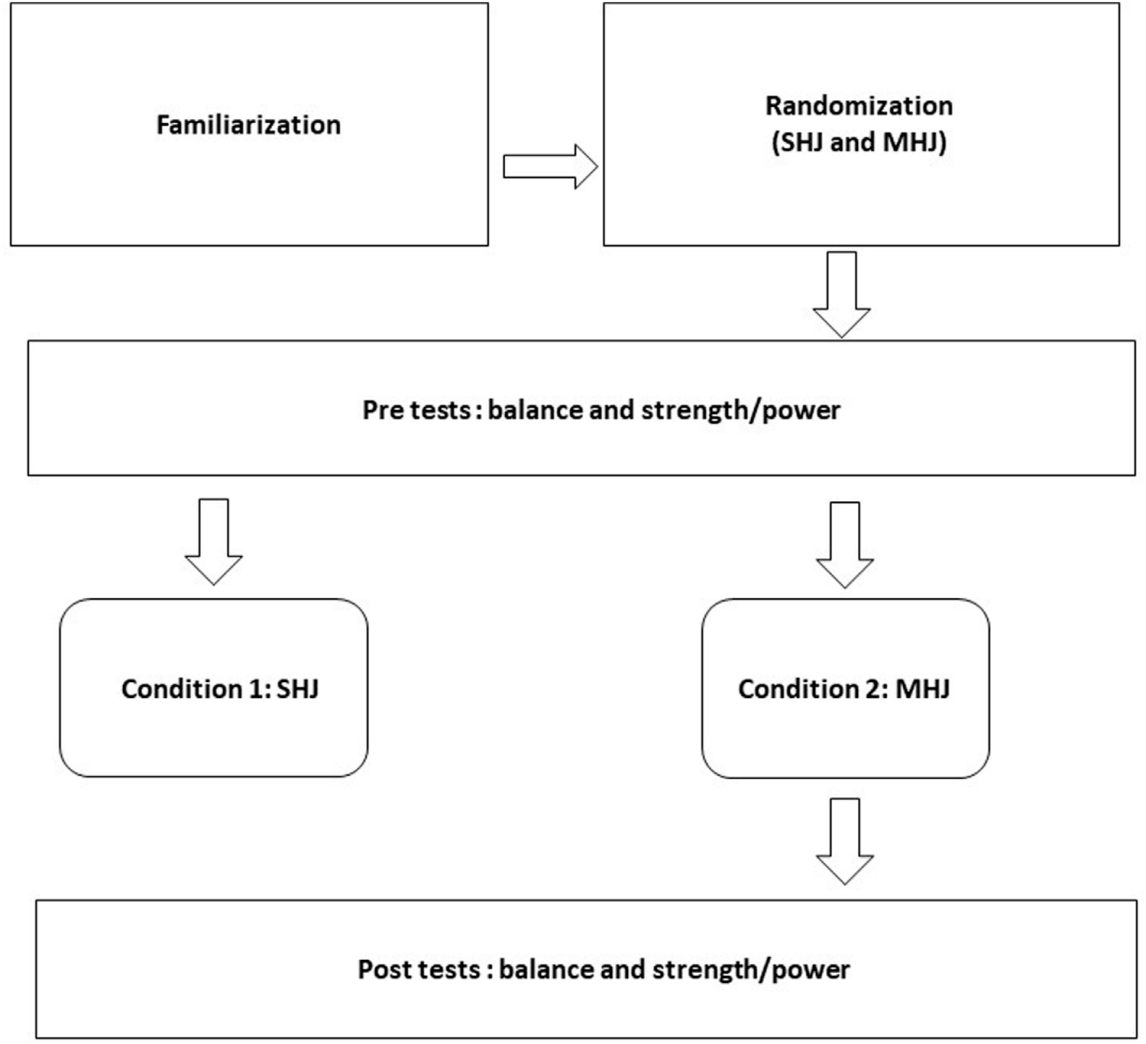
Study design. Notes: SHJ, submaximal hurdle jump exercises; MHJ, maximal hurdle jump exercises.

**FIGURE 2:**
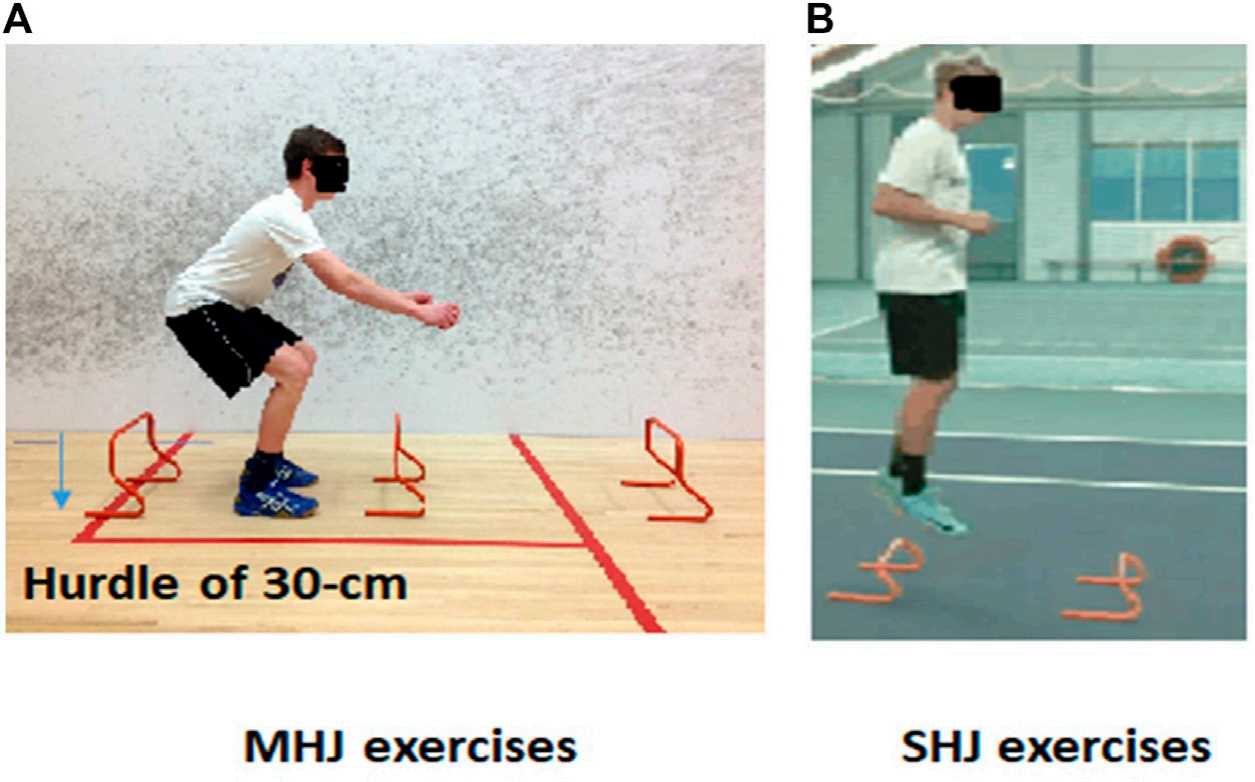
Schematic description of the two hurdle jump exercise protocols. **(A)** maximal hurdle jump exercise protocol (MHJ), **(B)** submaximal hurdle jump exercise protocol (SHJ).

Each plyometric exercise protocol (SHJ and MHJ) lasted 15-min and included a 10-min warm-up (Figure 1). MHJ comprised 3 sets of 6 maximal hurdle jumps over 30 cm. The MHJ involved the participants lowering themselves from an upright standing position until approximating a knee angle of 90°, followed immediately by a bilateral vertical countermovement jump over a hurdle of 30-cm of height (Figure 2A).

SHJ consisted of 3 sets and 6 repetitions of maximal side to side cone jumps. Participants were asked to hop bilaterally a submaximal side to side cone jumps (Figure 2B). The SHJ is considered a low- to moderate-intensity consecutive exercise (Lees et al., 2004). The two hurdle jump exercise protocols differed in terms of the maximal hurdle height. For both protocols, participants were asked to maximize their jump height on each repetition and minimize ground contact time. The rest between sets was approximately 2 min of recovery. Each session began with a standardized warm-up lasting approximately 10 min. The warm-up comprised submaximal intensity running, dynamic stretching, calisthenics, and preparatory jump exercises. Participants were also instructed to refrain from any strenuous activities before the test sessions. To minimize confounding factors, instructions related to sleep and diet were given to all athletes before the experiment started.

## Statistical analyses

All data analyses were performed using SPSS 26.0 (SPSS, Inc., Chicago, IL, United States). The level of significance was set *a priori* at *p* ≤ 0.05. Data were tested and confirmed for normal distribution using the Shapiro-Wilk test. Subsequently a 2 (condition: maximal, and sub-maximal plyometric conditions) × 2 (time: pre, post) analysis of variance (ANOVA) was computed with repeated measures on time to establish the acute effects of the different plyometric conditions on measures of balance and power performance. If condition × time interactions reached the level of significance, post-hoc tests (i.e., paired sample *t*-tests) were computed to identify the comparisons that were statistically significant. Additionally, effect sizes were determined by converting partial eta-squared from the ANOVA output to Cohen’s d (Cohen, 1988). Cohen’s d was classified as small (0.00 ≤ d ≤ 0.49), medium (0.50 ≤ d ≤ 0.79), and large (d ≥ 0.80). Test re-test reliability of the variables was assessed using Cronbach’s model of ICCs and standard error of measurements (SEM) according to the method of Hopkins et al. (2009). The level of significance was set at *p* < .05. The statistical analysis was carried out using IBM SPSS (version 25).

## Results

All 30 volleyball players completed the study. Participants attended all exercise and test sessions, and none reported any exercise- or test-related injury. Table 2 displays the test–retest reliability analyses for all applied tests. ICCs showed good reliability for all balance and power parameters and ranged from 0.81 to 0.89, with a SEM from 0.97 to 7.81. Furthermore, a paired *t*-test showed no significant differences between the scores recorded during the two trials for all measured variables.

### Postural balance

**FIGURE 3:**
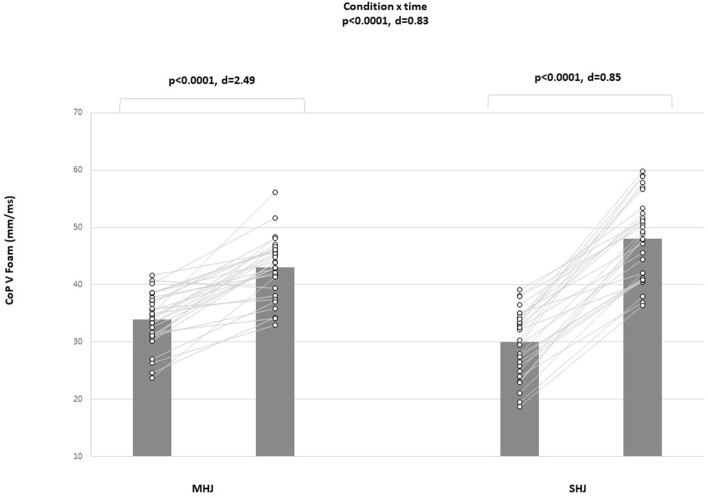
Mean values (black) and individual scores (white) for center of pressure velocity on foam surface (CoP V Foam) pre- and post-intervention; d, Cohen’s d; MHJ, maximal hurdle jump; SHJ, submaximal hurdle jump.

The CoP SA firm, the CoP SA foam, the CoP V firm, and the CoP V foam displayed significant condition-by-time interactions (all *p* < .0001; d = 0.82, 0.82, 0.85 and 0.83, for the CoP SA firm, CoP SA foam, CoP V firm, and CoP V foam, respectively) (Figure 3). Post-hoc analyses showed significant improvements in all balance variables from pretest to posttest in favor of the maximal plyometric condition (Table 3).

**TABLE 3:** Acute effects of maximal versus sub-maximal hurdle jump exercise protocols on measures of balance, vertical jump, reactive strength and leg stiffness in male youth volleyball players (means ± standard deviations).

Variables	Groups	Pre-intervention	Post-intervention	Change %	ANOVA *p*-value (Cohen’s d)			
					Cohen’s d	Time	Condition	Condition x time
Balance performance								
CoP SA firm	SHJ	1355.90 ± 403.61	820.08 ± 373.64	39.52	1.82 [Large]	0.0001 (2.12)	0.009 (0.49)	0.0001 (0.80)
	MHJ	1851.64 ± 486.37	714.23 ± 315.13	61.43	3.46 [Large]			
CoP SA foam	SHJ	1478.13 ± 457.43	986.47 ± 393.25	33.26	1.53 [Large]	0.0001 (2.00)	0.05 (0.36)	0.0001 (0.82)
	MHJ	1979.59 ± 491.34	793.14 ± 351.15	61.43	3.40 [Large]			
CoP V firm	SHJ	70.26 ± 16.41	49.13 ± 12.30	30.08	2.41 [Large]	0.0001 (2.58)	0.001 (0.60)	0.0001 (0.85)
	MHJ	87.98 ± 10.22	46.13 ± 9.39	47.57	3.71 Large]			
CoP V foam	SHJ	68.49 ± 13.79	48.95 ± 9.40	28.53	2.47 [Large]	0.0001 (2.49)	0.0001 (0.85)	0.0001 (0.83)
	MHJ	88.33 ± 12.72	49.29 ± 11.35	79.22	4.09 [Large]			
Power performance								
CMJ (cm)	SHJ	21.12 ± 3.91	22.55 ± 4.04	6.77	1.19 [Large]	0.0001 (0.83)	0.001 (0.70)	0.023 (0.42)
	MHJ	22.10 ± 3.27	26.60 ± 3.23	20.34	1.95 [Large]			
RSI (mm/ms)	SHJ	21.12 ± 4.04	22.55 ± 4.10	55.93	1.18 [Large]	0.0001 (3.14)	0.0001 (1.44)	0.0001 (1.58)
	MHJ	0.58 ± 0.13	1.62 ± 0.22	180.46	5.12 [Large]			
Leg stiffness (KN/m/Kg)	SHJ	29.99 ± 5.67	48.00 ± 7.06	60.08	3.09 [Large]	0.0001 (2.41)	0.630 (0.08)	0.0001 (0.78)
	MHJ	33.91 ± 4.61	43.07 ± 5.24	27.03	1.77 [Large]			

Notes: Values are expressed as means and standard deviations (SD), CI, 95% confidence limits; SHJ, submaximal hurdle jump exercise; MHJ, maximal hurdle jump exercise; CoP SA, center of pressure surface area; CoP V, center of pressure velocity; CMJ, countermovement jump; RSI, strength reactive index.

### Muscle power

**FIGURE 4:**
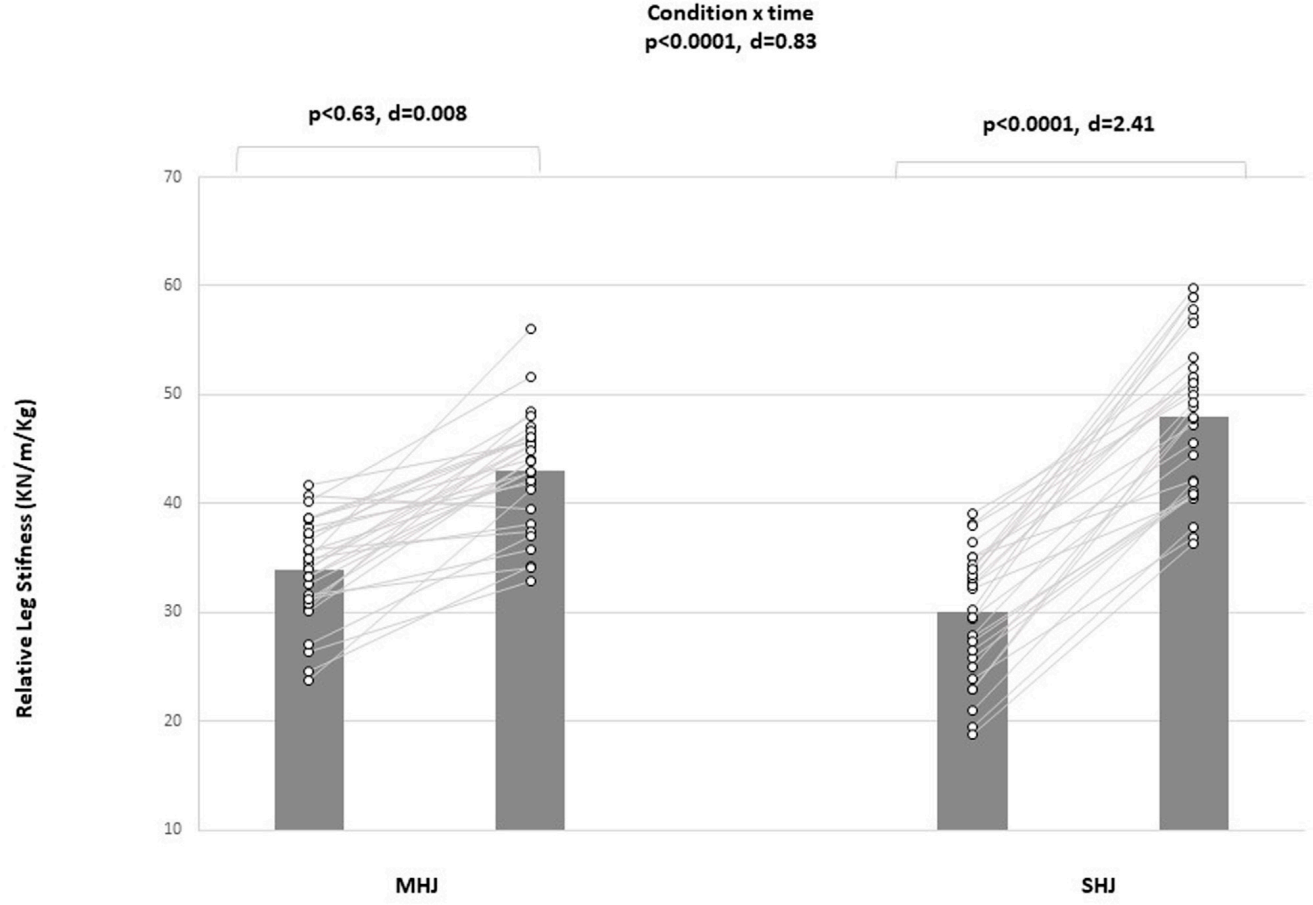
Mean values (black) and individual scores (white) for relative leg stiffness pre- and post-intervention; d, Cohen’s d; MHJ, maximal hurdle jump; SHJ, submaximal hurdle jump.

All the power tests displayed significant condition-by-time interactions [CMJ (d = 0.42, *p* < .05), RSI (d = 1.58, *p* < .0001), leg stiffness (d = 0.78, *p* < .001)] (Figure 4). Post-hoc analyses showed significant improvements in CMJ, and RSI performance from pretest to post test in favor of the maximal plyometric condition [CMJ (*p* < .001, d = 1.95); RSI (*p* < .001, d = 5.12)]. The post-hoc analysis showed a significant improvement (*p* < 0.001; d = 3.09) for leg stiffness in favor of the sub-maximal plyometric condition (Table 3).

## Discussion

This study examined the effects of different hurdle jump exercise protocols, involving maximal and submaximal hurdle jumps (MHJ and SHJ), on balance, RSI, vertical jump performance and leg stiffness in youth volleyball players. The results of the current study add insight for coaches and athletes in regards of using plyometric exercise training in youth athletes for subsequent performance enhancement. With regards to the present hypothesis, the main result was that MHJ demonstrated greater benefit for balance and reactive strength and vertical jump height performance as the MHJ tasks are highly dynamic compared with SHJ. However, the SHJ only enhanced leg stiffness. Findings from this study suggest that youth volleyball players a single session of MHJ exercise improved their balance, reactive strength and vertical jump performance. However, if the goal is to improve leg stiffness, SHJ exercises may offer greater benefit than MHJ exercises.

Our results indicate a significant, acute effect of MHJ on balance performance compared to SHJ in youth volleyball players. Using maximal plyometric action, the specificity of the exercises induce a training stress on stability (Lauber et al., 2021; Werfelli et al., 2021). In respect to balance performance the results of the present study are not unexpected. Jump exercise can significantly enhance postural control by promoting anticipatory postural adjustments (Gantchev and Dimitrova, 1996). Such anticipatory postural adjustments have been considered to occur in the peripheral joints and during maximal exercises and repeated exposure to stability and postural control challenges leads to feedforward adjustments that would activate appropriate muscles prior to landing (Marigold and Patla, 2002; Paillard et al., 2005). It can therefore be argued that the sensitivity of the afferent feedback pathway is greater following MHJ exercises than SHJ (Borghuis et al., 2008). Although maximal jump exercise (i.e., MHJ) is prevalent in volleyball training, we conceptualized that MHJ exercises were performed under less stable conditions with increased-speed, dynamic contractions performed within a smaller base of support or with the center of gravity being moved outside the base of support (Taube et al., 2008; Hammami et al., 2016; Lauber et al., 2021). As a consequence, MHJ exercise is likely to elicit a greater effect on balance performance than SHJ exercises. MHJ is considered a dynamic form of resistance training; (Komi et al., 1987; Kibele et al., 2014). Thus, MHJ-specific benefits on subsequent equilibrium were more evident that SHJ in the present study. Finally, it is important to note that the participants in the present study were all well-trained youth volleyball players, who already performed maximal jump exercises in their training sessions. Moreover, the SHJ exercises used in the present study comprised actions that were undertaken with a much lower amplitude compared to training loads regularly applied throughout the players’ volleyball training routine. Therefore, it is possible to argue that were of sufficient demand to challenge the study participants’ balance.

This study revealed that the MHJ exercises were successful in inducing significant acute effects on reactive (RSI) and CMJ performance in youth volleyball players. The effects observed in the current study are theoretically underpinned. Prior work by Lees et al. (2004a) demonstrated that maximal jumps are achieved through the greater engagement of the hip extensor muscles which leads to an increased tolerance to the eccentric loading placed on the musculotendon unit. More specifically, greater stretch-reflex contribution (Voigt et al., 1998), rate-of-force development (Matavulj et al., 2001), and increased desensitization of Golgi tendon organs (OVALLE, 1987; Hutton and Atwater, 1992), represent plausible neural mechanistic adaptations that enabled the participants to better tolerate and overcome impact forces experienced in the MHJ protocol. Similarly, given the acute nature of the present study, the activation of high threshold motor units or potentially an effect of post activation potentiation (Sale, 2002; Berriel et al., 2021), might also explain the positive acute adaptation displayed by the study participants for RSI and CMJ.

The results of this study also indicate that SHJ exercises have a favorable effect on leg stiffness in youth volleyball players. As, submaximal jumps appear to stress the ankle and knee muscles as adequately as maximal jumps (Lees et al., 2004a), it can be postulated that among the different types of plyometric exercises, submaximal exercises best mimic the demands of the subsequent leg stiffness performance (Lloyd et al., 2012). The improvements in leg stiffness observed in the present study might be related to the significant reductions in ground contact time, suggesting that the SHJ exercises (less ground contact time) were successful in enhancing the rebounding properties of the musculotendon unit. In relation to the spring-mass model (McMahon and Cheng, 1990), decrements in ground contact time would require greater rate-of-force development and more effective use of elastic energy reutilization to maintain center of mass displacement. Additionally, previous research has identified that with reductions in ground contact times, there is a concomitant increase in stretch-reflex activity reliance (Moritani et al., 1991; Oliver and Smith, 2010; Lloyd et al., 2012) and that neural regulation of the leg extensors were strong predictors of leg stiffness in boys (Oliver and Smith, 2010; Lloyd et al., 2012). Hence, we can speculate that SHJ exercises enhanced the reflexive contribution to overall leg stiffness by increasing muscle activity before, and during, ground contact and with less contact time than MHJ.

This study is not without limitations. First, we examined a sample of youth volleyball players. Therefore, the results of this study are specific to the population under investigation. Second, we evaluated the effects of two different jump exercise on subsequent balance and muscle strength and power performance. However, we did not study the acute effects of these exercises on other fitness tests that are relevant for volleyball performance (i.e., change-of-direction and speed). Future studies should, therefore, examine the acute effects of plyometric exercises on other fitness capabilities, such as speed and CoD. Third, postural control was not tested under sport-specific situation in this study which may have prevented any observation of greater effects. Future studies should include balance tests during the performance of volleyball exercises. Fourth, we did not include a control condition in the current study. Consequently, the outcomes of the present study have to be interpreted with caution.

## Conclusion

The outcomes of this study show positive effects of MHJ versus SHJ exercise protocols on subsequent balance, RSI and vertical jump performance in youth volleyball players. The results of the present study should therefore be translated into regular volleyball training to benefit from success in competition and injury risk incidence. Coaches should be mindful of the results of the present study when working with youth volleyball players in plyometric training settings. From a practical standpoint, the positive acute effects of MHJ exercises on postural balance, reactive strength and vertical jump performance were more evident than submaximal plyometric exercises. Therefore, MHJ exercise appears to be more valuable as a pre-workout (pre-training) protocol than SHJ exercises if the objective is to enhance balance and muscle strength, power performance.

From a practitioner’s point of view, the most important outcome of this study is that MHJ is more effective than SHJ to improve measures of balance and muscle strength/power. Accordingly, maximal hurdle jump exercises should be preferred over submaximal exercises during the warm-up of volleyball training sessions in youth athletes. Three sets of 6 maximal hurdle jumps over 30 cm appear to be sufficient to induce acute performance enhancements. While our study sample consisted of rather experienced youth volleyball players, findings of this study should be compared with those from novice players. SHJ exercises could be more appropriate for less experienced volleyball players due to slower dynamics of the hurdle jump protocol. More research is needed in this area.

## Data Availability

The raw data supporting the conclusion of this article will be made available by the authors, without undue reservation.
